# Differences in oxidative stress profile in adolescents smoking waterpipe versus cigarettes: The Irbid TRY Project

**DOI:** 10.14814/phy2.14512

**Published:** 2020-07-30

**Authors:** Mahmoud A. Alomari, Karem H. Alzoubi, Omar F. Khabour

**Affiliations:** ^1^ Department of Physical Education Qatar University Doha Qatar; ^2^ Division of Physical Therapy Department of Rehabilitation Sciences Jordan University of Science and Technology Irbid Jordan; ^3^ Department of Clinical Pharmacy Jordan University of Science and Technology Irbid Jordan; ^4^ Department of Medical Laboratory Sciences Jordan University of Science and Technology Irbid Jordan

**Keywords:** cigarettes, oxidative stress, smoking, waterpipe

## Abstract

Waterpipe (Wp) tobacco smoking is widely spreading, especially among adolescents. It has also been associated with organ damage and disease risk resulting from oxidative stress. However, no studies have examined the effect of smoking Wp on oxidative stress among adolescents. In the current study, we evaluated serum levels of oxidative stress biomarkers and activities of antioxidant enzymes between adolescents smoking cigarette (Cg only), Wp only, both (CgWp) versus never. The results showed higher GSSG levels in the Cg only, Wp only, and CgWp versus the none group. Similarly, the activity of GPx was greater in the Cg only versus the Wp only and CgWp. The activity of catalase was higher in the Wp only/CgWp groups versus none. No main effect of smoking on GSH, or GSH/GSSH, SOD, or TBARS was detected. In conclusion, Wp smoking among adolescents is associated with induction of stress oxidative to a similar extend of Cg smoking. Interventions and studies for smoking cessation and harm reduction that target Wp smoking among adolescents are needed.

## Abstract 2

Waterpipe (Wp) tobacco smoking is widely spreading, especially among adolescents. In the current study, we evaluated serum levels of oxidative stress biomarkers and activities of antioxidant enzymes between adolescents smoking cigarette (Cg only), Wp smoking among adolescents is associated with induction of stress oxidative to a similar extend of Cg smoking. Interventions and studies for smoking cessation and harm reduction that target Wp smoking among adolescents are needed.

## 1 INTRODUCTION

Tobacco use is one of the most potent, prevalent (˃1.1 billion users), and addictive social habit that has dominated humanity behavior across the globe for centuries (Pratiti & Mukherjee, 2019; World Health Organization, 2020). Its effect is systematic, results in smoking‐related diseases, and remains the second cause of death with 9 million annual deaths (Pratiti & Mukherjee, 2019). Waterpipe (Wp) smoking is a tobacco consumption style that involves burning moasel/tobacco on a charcoal. The smoke from the burning tobacco is inhaled immediately with the smoker's mouth after passing through a bowl of water (Pratiti & Mukherjee, 2019). It is a social behavior proliferating throughout the globe. It's prevalence is increasing in Europe, North and South America, Australia, East Asia, Africa and the Middle East (Pratiti & Mukherjee, 2019). This increase is particularly soaring among adolescents (Al‐Sheyab, Alomari, Shah, Gallagher, & Gallagher, 2014; Othman, Aghamohammadi, & Nik Farid, 2019), with some countries showing ~ 60% prevalence in this vulnerable group (Alomari & Al‐sheyab, 2018b). Many factors have been implicated in the spread of Wp smoking among adolescents, however peer pressure, socializing, and health misconceptions seem to spur this habit. The Wp has been linked to many devastating diseases (Waziry, Jawad, Ballout, Al Akel, & Akl, 2017) including cardiovascular (Almedawar, Walsh, & Isma'Eel, 2016), metabolic (Ebrahimi et al., 2019; Pratiti & Mukherjee, 2019), and respiratory (Hawari et al., 2019) diseases, cancer (Montazeri, Nyiraneza, El‐Katerji, & Little, 2017), and stroke (El‐Hajj, Salameh, Rachidi, Al‐Hajje, & Hosseini, 2019).

Oxidative stress is a cellular imbalance between oxidants and antioxidants, resulting from a mismatch between free radical production and antioxidants utilization (Sies, 2015). Free radicals are highly unstable and reactive molecular species due to an unpaired electron in an atomic orbital. Given the imbalance and hyperactivity, these free radicals easily react with other molecules causing systematic cellular, organ, and tissue damage (Cabello‐Verrugio, Simon, Trollet, & Santibanez, 2017). Although the mechanism and role are yet to be precisely defined, this damage has been implicated in the risk, development, morbidity, and mortality of many systematic diseases. These systems include the cardiovascular (Senoner & Dichtl, 2019), pulmonary (van der Vliet, Janssen‐Heininger, & Anathy, 2018), metabolic (Ighodaro, 2018), immune (Klaunig, 2018), musculoskeletal (Ahmad, Ansari, & Haqqi, 2020), renal (Duni, Liakopoulos, Roumeliotis, Peschos, & Dounousi, 2019), and neurocognitive (Choi & Lee, 2017).

Increased oxidative stress has been attributed to many factors, including tobacco consumption. Cigarettes (Cg) (Napierala et al., 2019b) and Wp (Badran & Laher, 2020) smoking seems to elicit a redox of free radicals, which in turn activate and release proinflammatory factors into the circulation. Additionally, this increase in circulatory free radicals appears to deplete endogenous antioxidants. The increase in free radicals and depletion of antioxidants can result in oxidant/antioxidant imbalance, thus augmented oxidative stress. Few studies, however, examined the effect of tobacco consumption (Kahraman, Torun, Osmanoglu, Oruclu, & Ozer, 2017; Tobore, 2019) on oxidative stress among adolescents, with none using Wp. Greater total antioxidant status and total oxidant status, and lower paraoxonase‐1 were found in adolescent passive Cg (Kahraman et al., 2017) and e‐cigarettes (Tobore, 2019) smokers. This increase in oxidative stress was related to number of cigarette exposure (Yildirim, Sermetow, Aycicek, Kocyigit, & Erel, 2011) and resulted in DNA damage of the peripheral blood lymphocytes (Shermatov et al., 2012). However, no studies have examined the effect of smoking Wp on oxidative stress among adolescents. Given the spread of Wp smoking among adolescents and young adults and the importance of oxidative stress for health, finding the effect of Wp smoking on oxidative stress status would better help understanding disease risk and development among adolescents. The current study compares serum levels of reduced glutathione (GSH), oxidized glutathione (GSSG), GSH/GSSG, catalase, superoxide dismutase (SOD), glutathione peroxidase (GPx), and Thiobarbituric acid reactive substances (TBARS) in adolescents smoking Cg, Wp, CgWp (smoking both), versus never.

## 2 MATERIALS AND METHODS

### 2.1 Participants and design

The data for this study are derived from a larger project, the Irbid‐Try. The recruitment process that has previously been explained (Alomari & Al‐Sheyab, 2018a, 2018b; Alomari et al., 2018a, 2018b; Alomari, Al‐Sheyab, & Mokdad, 2020a; Al‐Sheyab & Alomari, 2018), however, are briefly described herein.

Using cross‐sectional design for comparison of serum levels of oxidative stress biomarkers and activities of antioxidant enzymes among adolescents smoking tobacco (Cg only, Wp only, and CgWp) versus those who are not using any form of tobacco (none). The protocol of this study was approved by the Institutional Review Board of Jordan University of Science and Technology and the Ministry of Education of Jordan. Assents were obtained from all study participants, whereas written informed consents were obtained from the parents. Socio‐demographic characteristics, patterns of tobacco smoking, and blood samples were obtained from all participants.

### 2.2 Smoking status

Tobacco smoking status was assessed using a validated survey in Arabic language (Brener et al., 2013; Mzayek et al., 2012). In this survey, the participants were asked if they ever used either Wp or Cg. They were also asked about their patterns of Wp and Cg smoking. Participants were considered current smokers of Wp only, if they are Wp smoking without any other tobacco product within the past month (Mzayek et al., 2012). This same definition applied to current Cg smokers, whereas those who indicated past month smoking of both Cg and Wp were defined as dual smokers. In addition, those who smoked at least once during the last week were considered as regular tobacco (Wp, Cg, or dual) smokers.

### 2.3 Blood sampling

Venous blood samples (3 ml) were obtained from the antecubital veins of the study participants’ using plain vacutainer tubes with clot activators. Using centrifugation at 1500× *g* serum was obtained, and was, then, aliquoted in several tubes and stored at −80°C until time for further analysis (Giavarina & Lippi, 2017; Kolk, van Hoof, & Fiedeldij Dop, 2000).

### 2.4 Oxidative stress profile

Levels of total glutathione, GSSG, TBARs, GPx, catalase, and SOD were measured in serum samples using commercially available kits as per manufacturer's instructions. For all assays, samples were run in duplicates and absorbance was measured spectrophotometrically using Epoch Microplate Spectrophotometer (Bio‐tek instruments, Highland Park, Winooski, USA).

*GPx assay*. Activity of GPx was measured using a kit obtained from Sigma‐Aldrich, (catalog number: CGP1, MI, USA). The assay is based on the oxidation of GSH to GSSG by GPx, which is then coupled to the recycling of GSSG back to GSH via glutathione reductase and NADPH. The decrease in NADPH therefore reflects GPx activity. Changes in the absorbance with time was measured at 340 nm.

*GSH and GSSG assay*. A glutathione assay kit obtained from Sigma‐Aldrich was used (catalogue number: 38185, MI, USA). The assay is based on the reaction of GSH uses a kinetic assay in which catalytic amounts (nmoles) of GSH cause a continuous reduction of 5,5’‐dithiobis(2‐nitrobenzoic acid) (DTNB) to TNB and the GSSG formed is recycled by glutathione reductase and NADPH. Samples were deproteinized with the 5% 5‐Sulfosalicylic acid solution and centrifugation to remove the precipitated protein. To quantify GSSH in the samples, a masking reagent (provided by the kit) to neutralize GSH in the samples was used. GSH was estimated in the samples by subtracting the total GSSG from the total GSH. Changes in the absorbance with time was measured at 412 nm.

SOD assay: A kit obtained from Sigma‐Aldrich was used to measure SOD activity (catalogue number: 19160, MI, USA). The assay involves the reaction of tetrazolium salt with superoxide anion to produce formazan dye. The rate of this reaction is linearly related to the xanthine oxidase activity, and is inhibited by SOD. Changes in the absorbance with time was measured at 450 nm.

#### 2.4.1 Catalase assay

A kit from Cayman Chem (Ann Arbor, MI, USA) was used to measure catalase activity. The assay is based on the reaction of catalase with methanol in the presence of H2O2. The product of the reaction (formaldehyde) is measured colorimetically with 4amino‐3‐hydrazino‐5‐mercapto‐1,2,3‐triazole. Changes in the absorbance with time was measure at 540 nm.

#### 2.4.2 TBARs assay

A kit from Cayman Chem (catalogue number: 10009055, Ann arbor, MI. USA) was used to measure TBARs in the serum samples. The assay is based on the reaction of MDA with TBA at 90–100°C/acidic condition to produce the MDA‐TBA adduct, which can be measured colorometrically at 550 nm.

### 2.5 Statistical analyses

SPSS software for Windows (version 22.0; Chicago, IL) was used for all statistical analyses. Data are expressed as means ± *SD* or percentage (%), and α was preset at a significance level of *p* < .05. ANOVA was used to compare oxidative stress indices according to smoking status (i.e. Cg only, Wp only, CgWp, versus none). Additional LSD post‐hoc comparisons were used to determine differences between specific groups.

## 3 RESULTS

### 3.1 Participants

A total of 2,691 adolescents participated in the project, of which 2,445 (90.08%) gave information about smoking and 440 (16.3%) gave blood samples for the current study. As in Table 1, a total of 349 (14.4%) adolescents gave concurrent smoking information and blood samples.

**TABLE 1:** The participants' demographic characteristics (*n* = 349)

Gender (% Girls)	54.5
Age (years, mean (*SD*))	14.8 (1.0)
Weight (kg, mean (*SD*))	55.2 (12.8)
Height (cm, mean (*SD*))	161.8 (8.9)
BMI (kg/m^2^, mean (*SD*))	21.9 (4.2)
Underweight (%)	3.7
Normal weight (%)	68.2
Overweight (%)	18.5
Obese (%)	9.7
Grade (%)	
7	13.6
8	20.7
9	37.2
10	28.5
Location (%)	
Rural	60.7
Urban	39.3
Smoking Status (%)	
Never smoked	33.5
Cigarette smokers	7.2
Waterpipe smokers	22.3
Dual Smokers	37.0

Abbreviations: %, percent; cm, centimeter; kg, kilogram; SD, standard deviation.

The participants’ demographics are shown in Table 1. The age range was 14–17 years attending 7th‐10th grade. Most of the participating adolescents were females (54.5%), attending the 9th grade (37.2%), smoke Cg and Wp concurrently (37.0%), and normal weight (68.2%).

### 3.2 Relationship of smoking with serum oxidative stress profile

**FIGURE 1:**
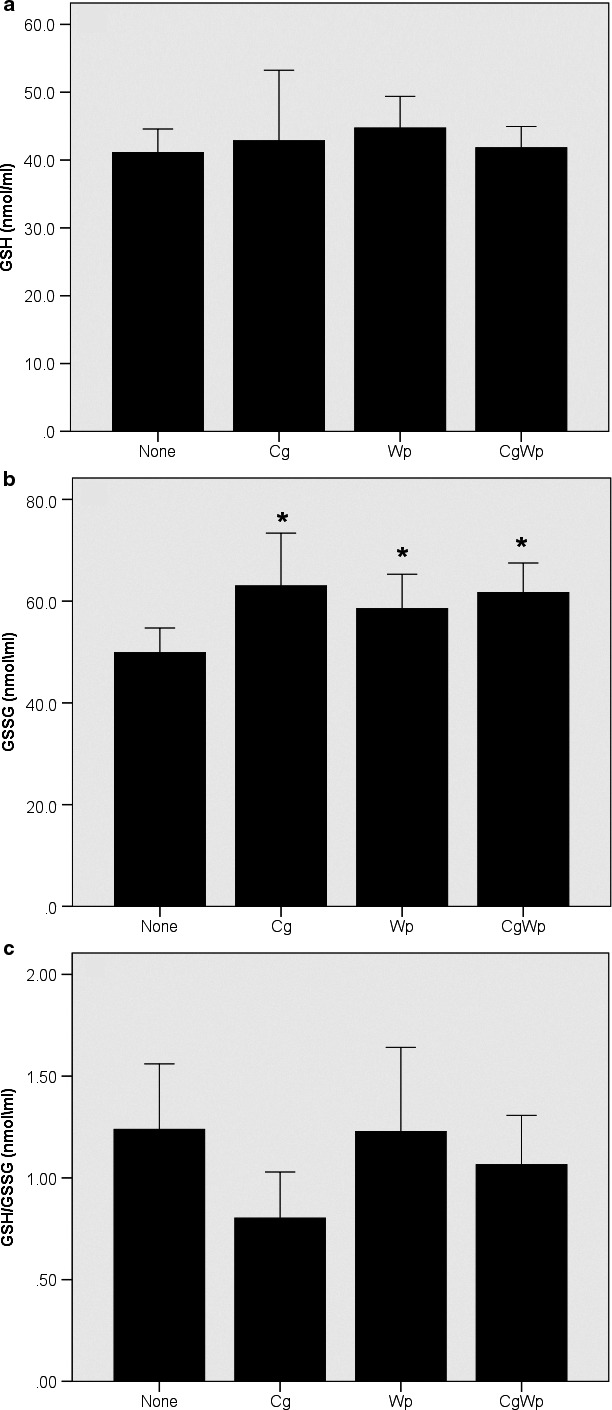
Differences in GSH (a), GSSG (b), and GSH/GSSG (c) in the smoking groups. The data are presented in mean ± SE. †: versus Cg smoking. *: versus nonsmoking using one way ANOVA followed by LSD post‐hoc comparisons

**FIGURE 2:**
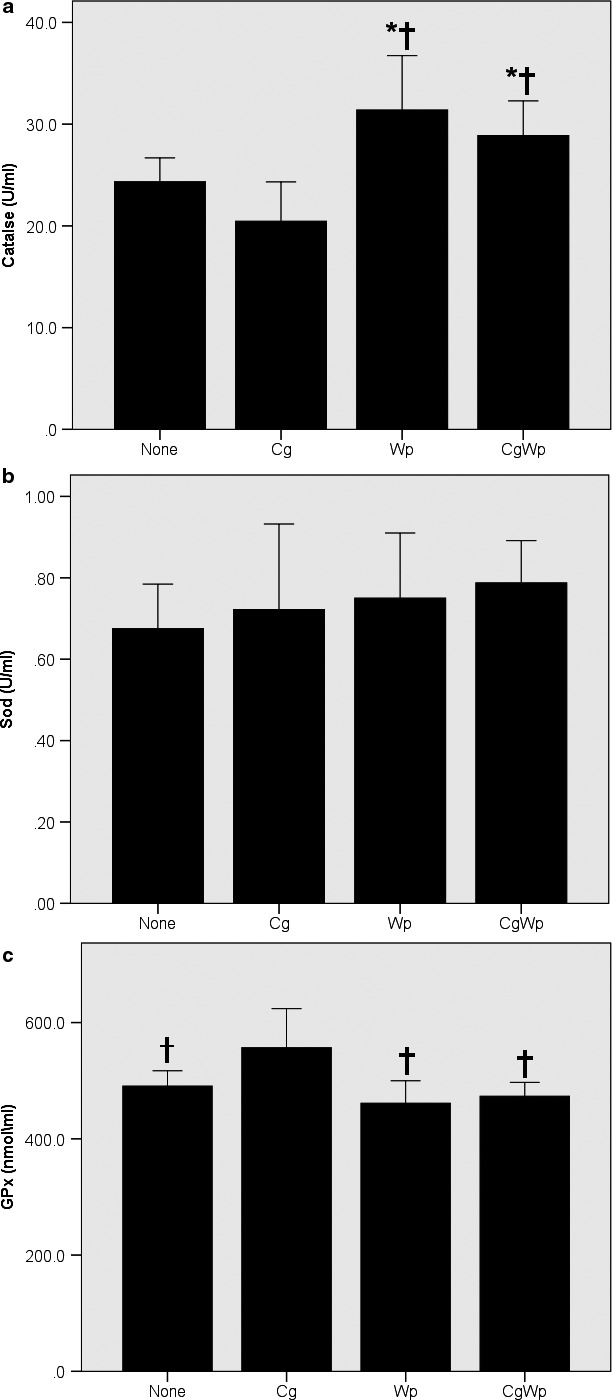
Differences in Catalase (a), Sod (b), and GPx (c) in the smoking groups. The data are presented in mean ± SE. †: versus Cg smoking. *: versus nonsmoking using one way ANOVA followed by LSD post‐hoc comparisons

**FIGURE 3:**
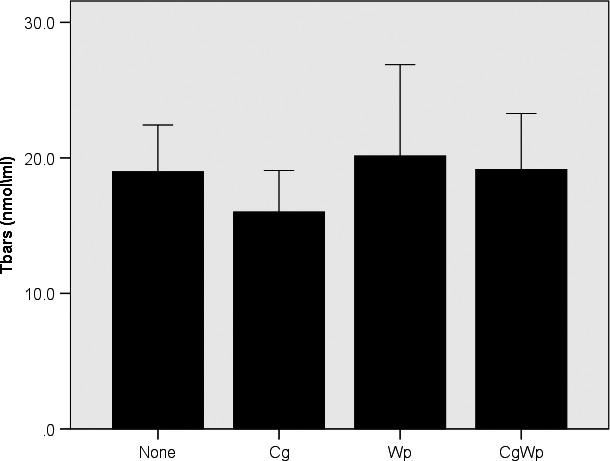
Differences in TBARS in the smoking groups. The data are presented in mean ± SE. †: indicate all groups versus adolescents smoking Cg using one way ANOVA followed by LSD post‐hoc comparisons

The ANOVA revealed a main effect of smoking for GSSG (*p* < .01), GPx (*p* < .03), and catalase (*p* < .009). Subsequent post‐hoc comparisons, shown in Figure 1b, revealed greater GSSG in the Cg only (*p* < .04), Wp only (*p* < .05), and CgWp (*p* < .002) versus the none group, without differences (*p* > .05) between the smoking groups (i.e. Cg only, Wp only, and CgWp). Similarly, as in Figure 2c, post‐hoc comparisons showed that GPx was greater in the Cg only versus the Wp only (*p* < .004), CgWp (*p* < .008), and the none groups, without differences (*p* > .05) between Wp only, CgWp, and none. Additionally, as Figure 2a depicts, the post‐hoc comparisons revealed greater catalase (*p* < .05) in the Wp only/CgWp groups versus the none and Cg only groups. No main (*p* > .05) effect of smoking for GSH, or GSH/GSSH (Figure 1) Sod (Figure 2), or TBARS (Figure 3).

## 4 DISCUSSION

The study examined the relationship of smoking with oxidative stress profile among adolescents. The results indicate altered glutathione and catalase in the adolescents smoking tobacco using either Cg, Wp, or both (CgWp).

Oxidative stress is an unhealthy condition that predisposes individuals to most fatal diseases including cardiovascular diseases and cancer (Liguori et al., 2018). Previous literature from human, animal and in *vitro* models provide strong evidence for the induction of oxidative stress by tobacco Cg smoking. In adult smokers, an increase in DNA oxidation (Mesaros, Arora, Wholer, Vachani, & Blair, 2012), lipid peroxidation, and alteration in catalase, SOD, GPx activity and glutathione level (Arja et al., 2013; Li et al., 2007; Miri, Saadati, Ardi, & Firuzi, 2012; Ozbay & Dulger, 2002) were observed in different body fluids when compared with nonsmokers. Exposure of pregnant women to active or passive Cg smoking induced oxidative stress in the placenta (Argalasova et al., 2019; Sbrana et al., 2011). In addition, lower concentrations of catalase and total antioxidative capacity were reported in the umbilical cord blood of smokers than nonsmokers (Aycicek & Ipek, 2008). Cg smoking during lactation has also been shown to induce oxidative stress in the mother's plasma, colostrum, and milk (Napierala et al., 2019a). This includes increases in TBARS and alterations in the activity of antioxidant (SOD, GST, GPx, and CAT) enzymes (Napierala et al., 2019a).

In children, active Cg smoking has been shown to induce oxidative stress as measured by the elevation of isoprostane in urine samples (Bono et al., 2019). Similarly, secondhand smoke was associated with elevated oxidative stress (Bono et al., 2014; Squillacioti et al., 2019) and subsequent induction of DNA damage (Chao et al., 2018) in adolescents. Using cultured mesenchymal cells, exposure to tobacco smoke induced oxidative stress by increasing superoxide radicals and reducing intracellular glutathione, CAT and glutathione reductase activity (Aspera‐Werz et al., 2018).

While the literature describing the effect of Cg smoking on oxidative stress is numerous, limited information is available on the relationship of Wp smoking with oxidative stress (Badran & Laher, 2020). Recent studies have shown similar effects of smoking Wp versus Cg. It has been shown that smoking Wp has induced oxidative stress, inflammation, tissue injury (Khan et al., 2020) and oxidative DNA damage (Alsaad et al., 2019) comparable to that of Cg smoking. This effect was further augmented when smoking Cg and Wp concurrently (Khan et al., 2020).

In animal models, exposure to Wp smoke has been shown to cause oxidative damage and subsequent tissue injury to various body organs including the lungs (Khabour et al., 2018; Khan, Sundar, & Rahman, 2018; Nemmar, Al‐Salam, Beegam, Yuvaraju, & Ali, 2020a), heart (Al‐Sawalha, Al‐Filali, Alzoubi, & Khabour, 2019a; Nemmar et al., 2020b), brain (Alqudah, Alzoubi, Ma'Abrih, & Khabour, 2018; Alzoubi, Halboup, Alomari, & Khabour, 2019a, 2019b), kidney (Al‐Sawalha, Alsari, Khabour, & Alzoubi, 2019c; Nemmar et al., 2020c; Rababa'h, Sultan, Alzoubi, Khabour, & Ababneh, 2016), liver (Charab, Abouzeinab, & Moustafa, 2016), and reproductive organs (Ali, 2017; Al‐Sawalha, Almahmmod, Alzoubi, Khabour, & Alyacoub, 2019b). These alterations include induction of oxygen‐reactive species, lipid peroxidation, and alteration of antioxidative enzyme activities (Badran & Laher, 2020).

The result of the current study is a pioneer in examining the impact of Wp smoking on oxidative status among adolescents. The results show altered glutathione oxidation‐reduction equilibrium and CAT and GPx activities in adolescents smoking Wp or Cg versus never smokers. The results also confirm the harmful effect of dual smoking (Wp and Cg) on adolescent health (Alomari et al., 2020a; Alomari, Al‐Sheyab, & Ward, 2018c; Alomari, Khabour, & Alzoubi, 2020b; Al‐Sheyab & Alomari, 2018).

The mechanisms by which Wp tobacco smoke can induce stress needs to be investigated. However, burned Wp tobacco and charcoal contain toxicants such as aromatic hydrocarbons, volatile aldehydes, nicotine, heavy metals, and others (El Hourani, Salman, Talih, Saliba, & Shihadeh, 2020; Jawad et al., 2019; Sepetdjian, Shihadeh, & Saliba, 2008), which can contribute to the observed induction of oxidative stress (Ito, Bekki, Fujitani, & Hirano, 2019; Reyes et al., 2013).

Tobacco‐induced oxidative stress contributes to several health effects that including pulmonary and cardiovascular diseases and cancer due to the degenerative processes in the exposed organs (Badran & Laher, 2020; Haddad et al., 2016). Accordingly, smoking including Wp, is expected to have long‐term effects on the adolescents’ future health (Alomari, Al‐Sheyab, Khabour, & Alzoubi, 2018a; Ramji et al., 2018).

Similar to Cg smoking, the current study indicates that Wp can induce oxidative stress. Therefore, harm reduction interventions targeting this population are essential. These interventions should aim at reducing levels of oxidative stress as shown by normalization of glutathione levels (Mons, Muscat, Modesto, Richie, & Brenner, 2016) as well as countering the adverse health effects of Wp smoking. Additionally, given the spiraling increase (Alomari & Al‐sheyab, 2018b), strategies to restrain Wp smoking among adolescents are needed. These strategies should include smoking cessation programs especially designed for Wp smoking among adolescents.

In the current study, not all oxidative stress biomarkers are examined. Direct measurement of reactive oxygen species in the smokers is strongly recommended. In addition, the study did not control for secondhand smoke exposure that might have an impact on the oxidative stress status among participants. Examining the impact of second hand Wp smoke exposure on body health is a matter of future studies. Longitudinal and intervention studies recruiting larger sample size from diverse populations are also warranted.

In conclusion, similar to Cg smoking, Wp smoking is associated with induction of oxidative stress in adolescents. Interventions and studies for smoking cessation and harm reduction that target Wp smoking among adolescents are needed.

## CONFLICT OF INTEREST

Author declare that they have no conflict of interest.

## AUTHORS CONTRIBUTIONS

MA conceived and designed the experiments; performed the experiments; analyzed and interpreted the data; and wrote the paper. OK has conceived and designed the experiments; analyzed and interpreted the data; and supported writing the paper. KA has helped in designing the experiments; performed the experiments; contributed reagents, materials, analysis tools or data; helped in writing the paper. All authors have approved the final version of the study.

## ETHICAL STATEMENT

The protocol of this study was approved by the Institutional Review Board of Jordan University of Science and Technology and the Ministry of Education of Jordan. Assents were obtained from all study participants, whereas written informed consents were obtained from parents.
